# Amplification of a Cytochrome P450 Gene Is Associated with Resistance to Neonicotinoid Insecticides in the Aphid *Myzus persicae*

**DOI:** 10.1371/journal.pgen.1000999

**Published:** 2010-06-24

**Authors:** Alin M. Puinean, Stephen P. Foster, Linda Oliphant, Ian Denholm, Linda M. Field, Neil S. Millar, Martin S. Williamson, Chris Bass

**Affiliations:** 1 Centre for Sustainable Pest and Disease Management, Rothamsted Research, Harpenden, United Kingdom; 2 Research Department of Neuroscience, Physiology, and Pharmacology, University College London, London, United Kingdom; Princeton University, Howard Hughes Medical Institute, United States of America

## Abstract

The aphid *Myzus persicae* is a globally significant crop pest that has evolved high levels of resistance to almost all classes of insecticide. To date, the neonicotinoids, an economically important class of insecticides that target nicotinic acetylcholine receptors (nAChRs), have remained an effective control measure; however, recent reports of resistance in *M. persicae* represent a threat to the long-term efficacy of this chemical class. In this study, the mechanisms underlying resistance to the neonicotinoid insecticides were investigated using biological, biochemical, and genomic approaches. Bioassays on a resistant *M. persicae* clone (5191A) suggested that P450-mediated detoxification plays a primary role in resistance, although additional mechanism(s) may also contribute. Microarray analysis, using an array populated with probes corresponding to all known detoxification genes in *M. persicae*, revealed constitutive over-expression (22-fold) of a single P450 gene (*CYP6CY3*); and quantitative PCR showed that the over-expression is due, at least in part, to gene amplification. This is the first report of a P450 gene amplification event associated with insecticide resistance in an agriculturally important insect pest. The microarray analysis also showed over-expression of several gene sequences that encode cuticular proteins (2–16-fold), and artificial feeding assays and *in vivo* penetration assays using radiolabeled insecticide provided direct evidence of a role for reduced cuticular penetration in neonicotinoid resistance. Conversely, receptor radioligand binding studies and nucleotide sequencing of nAChR subunit genes suggest that target-site changes are unlikely to contribute to resistance to neonicotinoid insecticides in *M. persicae.*

## Author Summary

*M. persicae* is the most economically important aphid pest in many regions of the world due to its large host range and the damage it causes through direct feeding and through the transmission of plant viruses. This species has developed resistance to most classes of insecticide; and although, to date, the neonicotinoids have remained an effective control measure, recent reports of resistance in *M. persicae* represent a threat to the long-term effectiveness of this chemical class. Work on other insect species has shown that resistance can arise through increased production of metabolic enzymes (such as cytochrome P450s) that detoxify the insecticide. In this study we made use of recent advances in genomics to quantify the expression of all genes encoding detoxification enzymes in a resistant strain of *M. persicae*. We found resistance correlated with the over-expression of a single P450 gene, and we show that this is due to gene amplification. We also showed that over-expression of cuticular proteins and reduced penetration of insecticide through the cuticle may also play a role in resistance. These findings will influence the design and monitoring of management strategies that aim to slow or prevent the development of resistance.

## Introduction

Insecticide resistance in crop pests has been a mounting constraint on crop protection since the introduction of synthetic insecticides in the 1940s, and has been shown to develop through three main mechanisms. These are 1) increased production of metabolic enzymes (such as esterases, glutathione transferases and P450-dependent monooxygenases) that break down or sequester the insecticide, 2) structural changes (mutations) in the gene encoding the insecticide target protein that make it less sensitive to the toxic effect of the insecticide (e.g. acetylcholinesterase for organophospates/carbamates, the voltage-gated sodium channel for pyrethroids, and the GABA receptor for cyclodienes) and 3) reduced penetration of insecticide through the cuticle [1].

The peach-potato aphid, *Myzus persicae* is an economically significant pest in many temperate regions of the world causing direct damage to a broad range of arable and horticultural crops and transmitting more than 100 plant viruses [2]. As a result this species has been treated extensively with insecticides and has developed multiple resistance to many classes of compounds including organophosphates, carbamates and pyrethroids. Three genetically independent mechanisms underlying resistance have been identified in *M. persicae*. Increased production of detoxifying carboxylesterases (E4 or FE4) confer broad-spectrum resistance to organophosphates and carbamates and results from gene amplification [3]. Two forms of target-site resistance have also evolved, a mutation in the gene encoding acetylcholinesterase (modified acetylcholinesterase, MACE) which results in insensitivity to dimethyl carbamates such as primicarb, and a mutation in the gene encoding the voltage-gated sodium channel (knockdown resistance, kdr) which confers resistance to pyrethroids [4]. As a result, neonicotinoids such as imidacloprid, clothianidin, thiamethoxam and thiacloprid, which are unaffected by these existing resistance mechanisms [5], are now the main class of insecticide used for *M. persicae* control. However, there have now been reports of resistance to the neonicotinoid imidacloprid in *M. persicae* from Europe, the USA and Japan [5]–[7], raising concerns for the long-term efficacy of this insecticide class.

Resistance to neonicotinoids is a significant problem in several insect species including the Colarado potato beetle (*Leptinotarsa decemlineata*), the brown planthopper (*Nilaparvata lugens*) and the tobacco whitefly (*Bemisia tabaci*) [5]. Neonicotinoids are nicotinic acetlycholine receptor (nAChR) agonists and resistance in a laboratory-selected colony of *N. lugens* was found to be conferred by a target-site modification (Y151S) within two alpha subunits of the nAChR [8]. However, to date, this remains the only example of target-site resistance and this mechanism is yet to be described in any field-collected insect population. Neonicotinoid resistance has been studied in greatest detail in *B. tabaci,* where resistance is associated with increased detoxification by P450s. Initially this detoxification mechanism was implicated by use of the P450 inhibitor piperonyl butoxide (PBO) and by biochemical assays with the model substrate 7-ethoxycoumarin [9]. Over-expression of a single P450 gene (*CYP6CM1*) has since been found to be associated with high levels of resistance to imidacloprid [10] and the role of this enzyme in resistance was demonstrated by studies of imidacloprid metabolism [11]. Over-expression of another P450 gene *CYP6G1* has also been associated with moderate imidacloprid resistance in the fruit fly *Drosophila melanogaster* and results from the insertion of an Accord transposable element into the 5′ end of the *CYP6G1* gene [12]. Although the mechanism(s) underlying neonicotinoid resistance in *M. persicae* have not been characterised there is evidence based on the differential effects of enzyme inhibitors that detoxification, via enhanced P450 activity, contributes to resistance [13].

P450s are a diverse class of enzymes with many functions ranging from biosynthesis to the metabolism of xenobiotics. Insect genomes have been found to contain from 46 to over 150 P450 genes, each encoding a different P450 enzyme [14], [15]. The rapid growth in genomics and post-genomic technologies has made it easier to study large and complex gene families such as the cytochrome P450 superfamily. Available resources include many full insect genome sequences, including the recently completed sequence of the pea aphid *Acyrthosiphon pisum* [16], expressed sequence tag (EST) libraries and tools such as microarrays and high-throughput transcriptome sequencing. Although the genome of *M. persicae* is yet to be sequenced, a number of other genomic resources are available. These include an EST library of 47,832 unique sequences and an oligonucleotide microarray populated with probes for nearly all available ESTs [17], [18]. A recent comparative analysis of detoxification enzymes in *A. pisum* and *M. persicae* revealed evidence of a substantial difference in the number of P450 genes in the two species, with this gene family at least 40% larger in *M. persicae*, where at least 115 unique P450 genes were identified [18]. In the work reported here, we have exploited the available genomic resources to investigate potential mechanisms of neonicotinoid resistance in a clone of *M. persicae* that shows 25–40 fold resistance to neonicotinoids.

## Results

### Topical bioassays

Four of five *M. persicae* clones (4106A, 4255A, 5142B and 1200Q) tested by a diagnostic dose bioassay (2.5 ng imidacloprid/aphid) showed complete susceptibility to imidacloprid with 100% mortality at the test dose. However, clone 5191A showed a significant decrease in mortality, with more than 50% surviving. The resistance profile of this clone was compared to the reference susceptible clone (4106A) by full dose-response bioassays with four neonicotinoid compounds, with and without pre-treatment with the P450 inhibitor PBO (see Table 1). Significant differences in LC_50_ values (the concentration required to kill 50% of the aphids tested) between 5191A and 4106A were observed for all of the insecticides tested, with the 5191A clone having a resistance factor between 27 and 41. Pre-treatment with PBO substantially synergised the effect of all of the insecticides tested, however, complete susceptibility was not restored with a resistance factor of 2.5–15 remaining.

**Table 1:** Results of full dose-response bioassays with a range of neonicotinoids to the *M. persicae* clones 4106A (susceptible) and 5191A (resistant) both with and without pre-treatment with the P450 inhibitor PBO.

Treatment	Clone	LC_50_ (mg l^−1^)	95% CL	Slope(±SE)	RF	SF
Imidacloprid	4106A	1.13	0.9–1.4	2.11(±0.21)		
Imidacloprid	5191A	31.1	7–69.7	0.69 (±0.10)	27.5	
Imidacloprid +PBO	4106A	0.1	0.07–0.15	1.64(±0.21)		11.3
Imidacloprid +PBO	5191A	1.55	0.56–2.55	1.82(±0.35)	14.86	20
Clothianidin	4106A	0.59	0.47–0.71	3.87(±0.58)		
Clothianidin	5191A	17.77	10.24–26.14	1.27 (±0.16)	30	
Clothianidin+PBO	4106A	0.24	0.19–0.31	2.65(±0.4)		2.46
Clothianidin+PBO	5191A	0.62	0.45–0.82	2.65(±0.34)	2.53	28.66
Thiametoxam	4106A	0.65	0.54–0.79	2.67(±0.22)		
Thiametoxam	5191A	19.7	14.87–25.74	1.77(±0.15)	30.14	
Thiametoxam+PBO	4106A	0.35	0.16–1.29	1.04 (±0.16)		1.86
Thiametoxam+PBO	5191A	1.06	0.77–1.45	1.52(±0.15)	3.05	18.58
Thiacloprid	4106A	0.61	0.27–1.06	2.55 (±0.32)		
Thiacloprid	5191A	25.18	11.32–63.47	0.92 (±0.14)	41.31	
Thiacloprid+PBO	4106A	0.23	0.13–0.39	1.45 (±0.19)		2.65
Thiacloprid+PBO	5191A	1.15	0.66–2.11	1.13(±0.15)	4.93	21.89

### Microarray analysis

Microarray analysis identified 273 genes significantly differentially transcribed between the insecticide resistant clone 5191A and the susceptible clone 4106A. The full list of these genes along with Log_2_, calculated fold-change values and a description based on the closest BLAST hit is given in Table S1. 174 genes (86 of unknown function) had elevated expression in the 5191A clone and 93 (55 of unknown function) were under-transcribed in this clone relative to 4106A. Of the 88 over-expressed genes with a known function, half (44) were potential candidates for being involved in insecticide resistance and are shown in Table 2. These included genes encoding cytochrome P450s, carboxylesterase E4/FE4 and a large number of cuticular proteins.

**Table 2:** Selected genes identified by microarray as significantly differentially transcribed between the insecticide resistant *M. persicae* clone 5191 and the susceptible strain 4106a.

Description	Mean Log2	Fold change	Parent sequence ID	Probe name
ref|X74555.1| M.persicae mRNA for esterase FE4	5.98	63.10	contig3118	M_persicae3118a
ref|X74554| M.persicae mRNA for esterase E4	4.16	17.89	contig720	M_persicae720a
ref|X74554| M.persicae mRNA for esterase E4	4.07	16.77	contig4586	M_persicae4586a
ref|X74555.1| M.persicae mRNA for esterase FE4	4.44	21.78	454Myzus_30192	CUST_8482_PI410703081
ref|X74555.1| M.persicae mRNA for esterase FE4	3.74	13.35	454Myzus_57259	CUST_15734_PI410703081
ref|XM_001948453.1| PREDICTED: Acyrthosiphon pisum similar to cytochrome P450 CYP6AX1 protein	2.93	7.63	contig749	M_persicae749b
ref|XM_001948453.1| PREDICTED: Acyrthosiphon pisum similar to cytochrome P450 CYP6AX1 protein	2.91	7.50	contig749	M_persicae749a
ref|XM_001948453.1| PREDICTED: Acyrthosiphon pisum similar to cytochrome P450 CYP6AX1 protein	2.41	5.30	contig497	M_persicae497a
ref|XM_001948453.1| PREDICTED: Acyrthosiphon pisum similar to cytochrome P450 CYP6AX1 protein	2.66	6.34	contig497	M_persicae497b
ref|XM_001948453.1| PREDICTED: Acyrthosiphon pisum similar to cytochrome P450 CYP6AX1 protein	2.42	5.37	contig5173	M_persicae5173a
ref AJ310563.1 Myzus persicae p450, Cyp6-5 like A. pisum CYP6AX1	2.77	6.81	AJ310563.1	CUST_18_PI304496669
ref|XM_001948453.1| PREDICTED: Acyrthosiphon pisum similar to cytochrome P450 CYP6AX1 protein	2.50	5.64	454Myzus_77428	CUST_29688_PI410703081
ref |DQ108939.1| Myzus persicae RR1 cuticle protein 2	1.49	2.82	contig2236	M_persicae2236a
ref |DQ108939.1| Myzus persicae RR1 cuticle protein 2	1.55	2.94	454Myzus_20621	CUST_589_PI410703081
ref|XM_001945378.1| PREDICTED: Acyrthosiphon pisum similar to Endocuticle structural glycoprotein SgAbd-9	1.32	2.50	contig1252	M_persicae1252a
ref|XM_001950803.1|Acyrthosiphon pisum similar to cuticular protein 111, RR-3 family (AGAP006931-PA)	2.06	4.17	454Myzus_63720	CUST_20651_PI410703081
ref|XM_001950771.1|Acyrthosiphon pisum similar to cuticular protein 111, RR-3 family (AGAP006931-PA)	1.83	3.56	contig10036	M_persicae10036a
ref|XM_001950771.1|Acyrthosiphon pisum similar to cuticular protein 111, RR-3 family (AGAP006931-PA)	1.92	3.78	454Myzus_23934	CUST_3815_PI410703081
ref|XM_001950771.1|Acyrthosiphon pisum similar to cuticular protein 111, RR-3 family (AGAP006931-PA)	1.73	3.32	454Myzus_23912	CUST_3794_PI410703081
ref|XM_001950771.1|Acyrthosiphon pisum similar to cuticular protein 111, RR-3 family (AGAP006931-PA)	1.84	3.59	contig7126	M_persicae7126a
ref|XM_001950771.1|Acyrthosiphon pisum similar to cuticular protein 111, RR-3 family (AGAP006931-PA)	1.56	2.96	454Myzus_21703	CUST_1641_PI410703081
ref|XM_001950771.1|Acyrthosiphon pisum similar to cuticular protein 111, RR-3 family (AGAP006931-PA)	2.85	7.22	454Myzus_68540	CUST_23572_PI410703081
ref|XM_001948091.1|Acyrthosiphon pisum similar to cuticular protein CPG12 (LOC100162620)	1.86	3.64	454Myzus_84753	CUST_34555_PI410703081
ref|XM_001951038.1|Acyrthosiphon pisum similar to cuticular protein CPG12 (LOC100162620)	2.25	4.76	454Myzus_23566	CUST_3455_PI410703081
ref|NM_001162838.1|Acyrthosiphon pisum similar to GA21525-PA (Acypi009812) Insect cuticle protein	1.28	2.42	454Myzus_31162	CUST_9030_PI410703081
ref|NM_001162838.1|Acyrthosiphon pisum similar to GA21525-PA (Acypi009812) Insect cuticle protein	1.07	2.10	454Myzus_30195	CUST_8485_PI410703081
ref|NM_001162838.1|Acyrthosiphon pisum similar to GA21525-PA (Acypi009812) Insect cuticle protein	1.15	2.22	454Myzus_79654	CUST_31316_PI410703081
ref|NM_001162838.1|Acyrthosiphon pisum similar to GA21525-PA (Acypi009812) Insect cuticle protein	1.11	2.16	454Myzus_23997	CUST_3875_PI410703081
ref|NM_001162295.1 Acyrthosiphon pisum similar to cuticular protein (Acypi008570)	3.40	10.57	454Myzus_70832	CUST_25035_PI410703081
ref|NM_001162295.1 Acyrthosiphon pisum similar to cuticular protein (Acypi008570)	2.64	6.24	454Myzus_72563	CUST_26222_PI410703081
ref|NM_001162295.1 Acyrthosiphon pisum similar to cuticular protein (Acypi008570)	3.48	11.16	454Myzus_77060	CUST_29423_PI410703081
ref||XM_001949737.1| PREDICTED: Acyrthosiphon pisum similar to cuticular protein CPG12	2.04	4.10	454Myzus_72318	CUST_26042_PI410703081
ref||XM_001949737.1| PREDICTED: Acyrthosiphon pisum similar to cuticular protein CPG12	3.16	8.92	454Myzus_21883	CUST_1818_PI410703081
ref|XM_001950188.1| PREDICTED: Acyrthosiphon pisum similar to tentative cuticle protein	2.98	7.90	contig3580	M_persicae3580a
ref|XM_001950188.1| PREDICTED: Acyrthosiphon pisum similar to tentative cuticle protein	3.00	7.98	454Myzus_23194	CUST_3095_PI410703081
ref|XM_001950188.1| PREDICTED: Acyrthosiphon pisum similar to tentative cuticle protein	1.68	3.20	contig4585	M_persicae4585a
ref|XM_001950188.1| PREDICTED: Acyrthosiphon pisum similar to tentative cuticle protein	1.43	2.69	454Myzus_21311	CUST_1254_PI410703081
ref|NM_001162623.1| Acyrthosiphon pisum cuticle protein-like	1.80	3.48	454Myzus_52388	CUST_12053_PI410703081
ref|NM_001162623.1| Acyrthosiphon pisum cuticle protein-like	1.45	2.74	454Myzus_83261	CUST_33856_PI410703081
ref|XM_001945198.1| PREDICTED: Acyrthosiphon pisum similar to tentative cuticle protein	1.77	3.41	contig3047	M_persicae3047a
ref|XM_001950188.1| PREDICTED: Acyrthosiphon pisum similar to tentative cuticle protein	2.23	4.70	454Myzus_28670	CUST_7817_PI410703081
ref|XM_001945198.1| PREDICTED: Acyrthosiphon pisum similar to tentative cuticle protein	2.32	5.01	454Myzus_58311	CUST_16524_PI410703081
ref|XM_001950188.1| PREDICTED: Acyrthosiphon pisum similar to tentative cuticle protein	2.44	5.42	454Myzus_30744	CUST_8769_PI410703081
ref|XM_001950188.1| PREDICTED: Acyrthosiphon pisum similar to tentative cuticle protein	2.61	6.11	454Myzus_67956	CUST_23268_PI410703081

Five EST sequences encoding carboxylesterase FE4 and the closely related variant E4 were identified as being over-expressed, in the resistant clone. However the level of expression between these sequences was variable (ranging from 13–63 fold). This variation probably resulted from the fact that only one of the five ESTs (contig 3118) was a perfect match with FE4 at the probe site, shown previously to be the variant present in 5191A [13]. Therefore, the fold-change indicated by the probe designed on contig 3118 is likely to be the most accurate and this level corresponds well with the 64-fold increase in the esterase level previously reported in aphids with the FE4 genes and R3 levels of resistance [19].

Five EST sequences (Table 2) encoding cytochrome P450s were elevated in the 5191A strain (5.3–7.6 fold). In two cases duplicate probes corresponding to the same EST sequence were identified (contig749a/b and contig497a/b). All five partial EST sequences were most similar to a single cytochrome P450 gene of *Acyrthosiphon pisum CYP6CY3* (77.3–88.7% at the nucleotide level) [15] and, as shown in Figure S1, align with different regions of the *A. pisum* gene and almost certainly correspond to the same gene in *M. persicae*.

Finally, a large number (32) of partial and full length EST gene sequences encoding cuticular proteins (CPs) were shown to be differentially transcribed between the 5191A and 4106A clones (Table 2). The changes in expression level of the 32 genes varied from 2.1–11.1 fold. The EST sequences on which these probes were designed vary considerably in length and it is difficult to ascertain the number of unique genes represented by these sequences. BLAST searching and a Hidden Markov Model tool on the CuticleDB WebSite (http://bioinformatics2.biol.uoa.gr/cuticleDB/index.jsp) [20] was used to characterise the different CPs shown to be over-expressed in the 5191A clone. All three members of the CPR group, as characterised by a conserved amino acid domain (the Rebers and Riddiford Consensus RR-1, RR-2 and RR-3), were represented along with several CPs of the cuticular protein glycine-rich (CPG) family.

Of the thirty eight genes of known function that were under-transcribed in 5191A relative to 4106A, only a limited number of detoxification genes were identified (see Table S1). These included four sequences encoding glutathione S-transferases (GSTs) with a negative fold change of -2 to -4 and a single sequence encoding a CYP6A2 type cytochrome P450 with a fold change of -6.4.

Real-time quantitative PCR was used to validate the microarray results by examining the expression profile of ten selected genes: the *CYP6CY3* gene and nine of the over-expressed genes encoding cuticular proteins. In all cases, the over-transcription of the genes was confirmed (Table 3), although, as reported previously for the Agilent array platform, expression ratios obtained from RT-PCR were frequently higher than those generated by the microarrays [21]. The *CYP6CY3* gene was found to be over-transcribed 22 fold (+/−0.91).

**Table 3:** Fold change in expression of *CYP6CY3* and cuticular proteins in the insecticide resistant *M. persicae* clone 5191 (compared to the susceptible reference clone 4106A) as determined by quantitative PCR.

Protein gene ID	Fold change - qPCR	95% confidence limits	Fold change - microarray
CYP6CY3	22.04	1.8	5.3–7.5
7126	3.86	0.46	3.59
4585	3.17	0.27	3.2
65840	3.93	0.34	7.22
2236	4.28	0.26	2.82
1252	1.83	0.14	2.5
23566	13.52	1.29	4.76
31162	2.98	0.72	2.42
72563	15.55	6.28	6.24
3580	7.99	2.61	7.9

For comparison the fold change measured by microarray is also shown.

### *CYP6CY3* gene copy number

Quantitative PCR was used to determine *CYP6CY3* gene copy number using genomic DNA as template. Data were normalised using three genes; *para* (present in two copies in diploid insect genomes), *ace* (present in four copies in *M. persicae*) and actin (thought to be multi-copy). The clone 5191A showed a fold change in copy number of 9.4 (+/−0.3), 9.2 (+/−0.4) and 9.2 (+/−0.4) using each gene to normalise respectively compared to the 4106A clone. The mean cycle threshold values of three biological replicates in quantitative PCR of the *CYP6CY3* and sodium channel genes in 4106A were essentially the same (CTs of 18.56 and 18.50 respectively) indicating that the diploid genome of this clone carries two copies of the *CYP6CY3* gene and this is amplified to ∼18 copies in the resistant clone 5191A.

### *CYP6CY3* cDNA characterization

**Figure 1:**
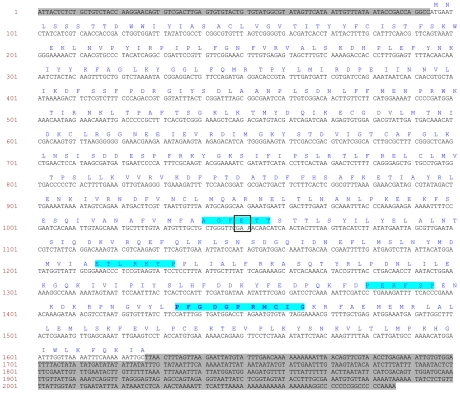
Complete mRNA sequence of *M. persicae CYP6CY3.* 5′ and 3′ untranslated regions are highlighted in grey. Sites of amino acid substitutions are boxed. Conserved domains common to cytochrome P450s or to CYP6 members are highlighted in blue such as the oxygen binding motif (position 316), the heme binding motif (position 447), the helix K motif (position 374) and a sequence motif (position 429) specific to CYP6 members.

The *CYP6CY3* sequence was initially derived from three non-overlapping EST sequences (479/5173 and 749 see Figure S1). The missing middle section and terminal 5′ UTR region of the gene were obtained by 5′ RACE and the complete mature mRNA sequence of 2087 bp is shown in Figure 1. This includes a 5′ UTR of 94 bp and a 3′ UTR of 460 bp. The cDNA has an open reading frame of 1533 bp encoding 511 amino acid residues. The predicted isoelectric point of the protein is 8.6 and the theoretical molecular weight is 59.13 kDa. *CYP6CY3* shares similarity with other microsomal proteins with a strongly hydrophobic N-terminus (resembling a signal peptide sequence) acting as a transmembrane anchor. As shown in Figure 1, the encoded protein contains conserved domains characteristic of P450s such as the oxygen binding motif (helix I) ([A/G]GX[E/D]T[T/S], position 316), the helix K motif (EXXRXXP, position 374), the heme-binding “signature” motif (PFXXGXXXCXG, position 447) and a sequence motif (PXXFXP, position 429) specific to CYP6 members.

**Figure 2:**
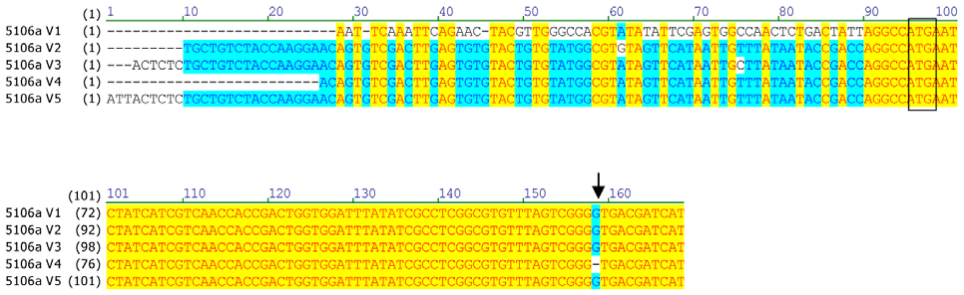
Alignment of the 5′UTR and N-terminal coding sequence of *CYP6CY3* copy variants in the *M. persicae* clone 5191a. The start codon is boxed. An indel which introduces a stop codon in copy variant 4 is indicated by an arrow.

Variation in the coding sequence of *CYP6CY3* copies in the two aphid clones was examined by both direct nucleotide sequencing and by cloning and sequencing the full cDNA ORF and/or 5′ RACE PCR products. In 4106A, direct sequencing revealed a very low level of variation in the sequence of the two copies with just one change (G/A) at position 319, indicating an E/K amino acid substitution. Interestingly, this change occurs in the oxygen binding motif, where the usual residue is either E or D (see above). However other insects also carry the K residue at this position with the *A. pisum* CYP314A1 and CYP314A2 P450s two examples. Attempts to direct sequence the *CYP6CY3* gene in the 5191A clone gave poor sequence quality (characterised by multiple overlaid sequences). To overcome this, PCR products of the full length cDNA coding sequence and 5′ RACE products were cloned and sequenced to look for sequence variation in coding sequence and 5′ UTR/promoter regions. In general this showed that the coding sequences of the *CYP6CY3* gene copies are conserved with only a single synonymous SNP observed in the full length cDNA clones. However, there were more differences in cloned 5 ′ RACE PCR products and an alignment of the 5′ UTR and N-terminal coding sequence of *CYP6CY3* copy variants (Figure 2) revealed one variant where the 5′ UTR/promoter region had been replaced. There was also evidence of at least one expressed pseudogene of *CYP6CY3* with a single base deletion which results in a frame shift and generates a stop codon one codon after the deletion (this frame shift variant probably explains the difficulty in obtaining clean sequence traces by direct sequencing). When the cDNA sequence of 4106A and 5191A are compared, only two completely conserved differences between the clones were observed, with 5191A having a synonymous (CGA/CGC) SNP at position 214 and a non-synonymous SNP (ACC/ATC) conferring a threonine to isoleucine substitution at position 24.

### Artificial feeding assays

Feeding the 5191A and 4106A *M. persicae* clones with insecticide using artificial feeding assays revealed significant differences in the calculated LC_50_ values compared to those derived from topical application. Differences were observed both between different neonicotinoids and between the two aphid clones (see Table 4). The LC_50_ in the feeding assays was 3.5 times lower for imidacloprid and 8.4 times lower for clothianidin and thiamethoxam for the susceptible clone than that calculated by topical bioassay. For the resistant clone the LC_50_ was 17 times lower for clothianidin. Unfortunately the LC_50_s for imidacloprid and thiamethoxam by feeding could not be calculated for the resistant clone because of anti-feedant effects (evident in the very shallow slope of the dose response line). As a consequence, the resistance ratio could only be calculated for clothianidin where it was approximately two-fold lower than calculated by topical bioassay. In all experiments the mortality of starvation controls was less than 10% when assessed at 48hs.

**Table 4:** Results of artificial feeding bioassays with a range of neonicotinoids to the *M. persicae* clones 4106A (susceptible) and 5191A (resistant).

Treatment	Clone	LC_50_ (mg l^**−**1^)	95% CL	Slope (±SE)	RF
Clothianidin	4106A	0.07	0.03–0.11	1.423 (±0.188)	
Clothianidin	5191A	1.02	0.62–1.81	1.037 (±0.158)	14.5
Imidacloprid	4106A	0.322	0.16–0.71	0.802 (±0.11)	
Imidacloprid	5191A	–	–	0.315 (±0.12)	–
Thiametoxam	4106A	0.077	0.01–0.23	1.043(±0.173)	
Thiametoxam	5191A	–	–	0.216(±0.152)	–

### Penetration of imidacloprid

**Figure 3:**
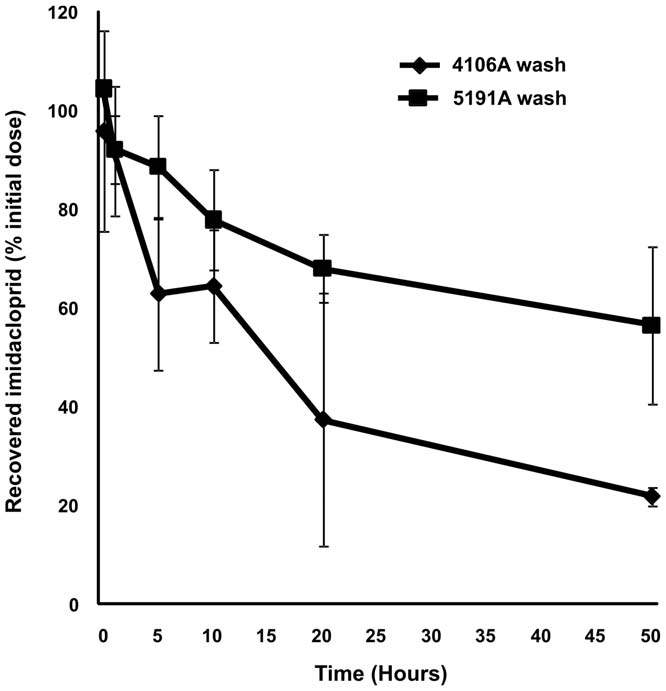
Result of *in vivo* penetration assay using [^3^H] imidacloprid on the 4106A (susceptible) and 5191A (resistant) clones. Graph shows the percentage of the initial [^3^H] imidacloprid dose recovered from the cuticle wash at different time points after application.

An *in vivo* penetration assay using [^3^H] imidacloprid revealed significant differences in the penetration of insecticide through the cuticle in the 4106A and 5191A *M. persicae* clones (see Figure 3). At the initial time points (30 min and 1 hour), the penetration of imidacloprid was relatively low, with no notable differences in the amount recovered by external rinsing between the susceptible and resistant clones. However, after 5 hours, the amount recovered from the susceptible clone had decreased sharply (to 63%), a trend that continued throughout the time course of the experiment, with only 22% of the initial imidacloprid dose recovered after 50 hours. In contrast, the levels of [^3^H] imidacloprid recovered from the resistant clone by external rinsing decreased at a much slower rate, with ∼88% of the applied dose being recovered after 5 hours and more than half the initial dose (56%) recovered after 50 hours, indicating a significant (p≤0.05) reduction in the rate of imidacloprid penetration through the cuticle compared to the susceptible clone. In future it may be interesting to relate how the penetration rate of imidacloprid observed in this study relates to the speed of action of this compound in resistant and susceptible clones (as determined by lethal time bioassays).

### [^3^H] imidacloprid binding assays

**Figure 4:**
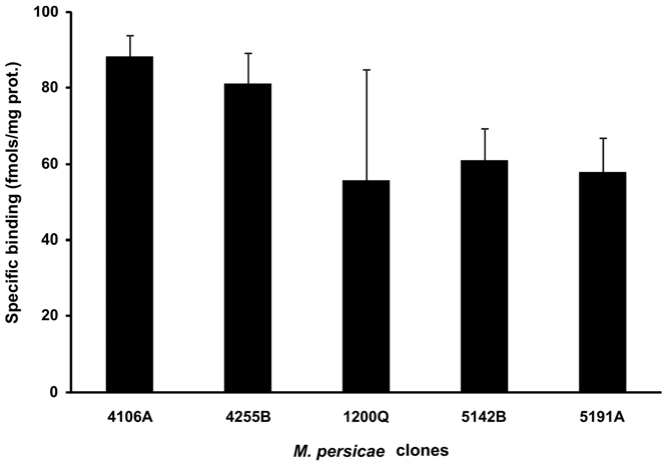
Specific [^3^H] imidacloprid binding to membranes extracted from a range of *M. persicae* clones. Bars represent 95% confidence limits.

Radioligand binding assays of [^3^H] imidacloprid to several independent membrane preparations from *M. persicae* indicated a high degree of variability in the levels of imidacloprid binding to the nicotinic acetylcholine receptor (nAChR) (Figure 4). Although there were significant differences in specific binding of [^3^H] imidacloprid between some of the clones analyzed, (e.g. between clones 5142B/5191A and clones 4106A/4255A), a consistent correlation with the resistant phenotype was not observed (see Figure 4), providing no evidence of target-site insensitivity in the 5191A clone.

### Sequence analysis of nAChR subunits

The N-terminal region of the *M. persicae* nAChR α1-α4 and β1 subunits, encompassing the conserved domains previously implicated in neonicotinoid binding (loops A to F) were amplified by PCR from several individuals of the 4106A and 5191A clones and examined by nucleotide sequencing. Although a limited number of silent SNPs were detected, no non-synonymous changes were observed and the deduced amino acid sequence in the region studied was identical.

## Discussion

Over-expression of one or more P450s currently appears to be, through direct or indirect evidence, the primary route for neonicotinoid resistance in insect pests. In the present study the involvement of P450s in neonicotinoid resistance in *M. persicae* was established by using the P450 inhibitor PBO, with a pre-treatment of PBO substantially synergising the effect of all the insecticides tested on a resistant *M. persicae* clone from Greece (5191A). A genomic approach was then used to quantify the expression of all known genes encoding detoxification enzymes in the resistant 5191A clone and a susceptible clone, 4106A, using a recently developed microarray populated with probes for >45 000 ESTs [17], [18]. A number of candidate genes with elevated expression were identified, including genes encoding a carboxylesterase, a P450 and several cuticular proteins.

The gene encoding the carboxylesterase FE4 was over-expressed 63-fold in the resistant clone, consistent with the 64-fold increase in the enzyme previously reported in aphids with FE4 genes at R_3_ levels [19]. A previous study examining the susceptibility of the 5191A clone to the pyrethroid α-cypermethrin reported a resistance factor of 33 to this compound which is similar to the level of resistance displayed by other aphid clones with the R3 level of esterase overproduction [13]. However, the enhanced level of carboxylesterase is unlikely to confer significant resistance to neonicotinoids since other *M. persicae* clones overproducing this enzyme show no resistance to imidacloprid [22].

A single P450 gene (represented by five ESTs) showed a 22-fold increased transcription in the resistant clone and the full-length cDNA sequence was most similar to a cytochrome P450 gene of *A. pisum* recently named as *CYP6CY3* [15]. The CYP6 family has been implicated in insecticide resistance more often than any other P450 family and the *M. persicae CYP6CY3* gene shares sequence identity with other *CYP6* P450 genes implicated in neonicotinoid resistance in *B. tabaci* and *D. melanogaster*. To date, the increased expression of P450 genes in resistant insects has been shown to mainly arise through mutations and indels in *cis*-acting promoter sequences and/or *trans*-acting regulatory loci [23]. In this study we show that the enhanced transcription of *M. persicae CYP6CY3* is due, at least in part, to amplification of the gene from 2 to ∼18 copies. Although duplication of P450 genes associated with insecticide resistance has been reported in *An. funestus* and *D. melanogaster* [24], [25] this study represents the first report of a P450 gene amplification event associated with insecticide resistance in an agriculturally important insect pest. In contrast, several species of crop pests including *M. persicae* have evolved resistance to insecticides through amplification of genes encoding esterases and glutathione-S-transferases [23]. There is also evidence from the recently sequenced *A. pisum* genome that aphids shows a propensity for gene duplication [16]. Amplification of esterase genes in *M. persicae* is linked to an autosomal 1,3 translocation event (*E4*) or involves multiple rearrangements (*FE4*) [4] but further work is required to characterise the mechanism by which the *CYP6CY3* is amplified. Sequencing of the *CYP6CY3* gene copies in the resistant strain showed a low level of variation in the coding sequence although there was evidence of one expressed pseudogene (caused by a single base deletion introducing a frame shift) and modification of the 5′ UTR sequence. Since the ∼9 fold increase in copy number may not fully explain the ∼22 fold increase in mRNA observed it is possible that additional mechanisms, such as upregulation of transcription, also contribute to over-expression of *CYP6CY3* in clone 5191A. The finding of at least one copy of *CYP6CY3* with a variant promoter sequence may be relevant and requires further investigation.

Although pre-treatment with PBO substantially synergised the effect of all the insecticides tested on the 5191A resistant clone, complete susceptibility was not restored (a resistance factor of between 2.5 and 15 remained) indicating that an additional mechanism(s) may play a role in the phenotypic resistance seen in this clone. However, the radioligand binding assays with [^3^H] imidacloprid to aphid membrane preparations and sequencing of the N-terminal (ligand binding) region of several nAChR subunits showed that target-site resistance is unlikely to be responsible. Conversely, the microarray analysis found a large number (32) of up-regulated sequences encoding cuticular proteins (CPs) in the resistant clone suggesting that a change in penetration of insecticide might be conferring some resistance. Although it is difficult to ascertain the exact number of unique genes represented by these ESTs (as many are short reads generated by 454 sequencing) all three members of the CPR group of CPs (the largest structural CP family in arthropods) were represented along with several CPs of the CPG family. Recent annotation of CPs in the pea aphid genome has revealed a family of 92 CPR genes [26]. Furthermore, in *A. pisum* CP genes were found to be clustered in the genome and may also be co-regulated [26] which may explain why several ESTs encoding CPs were identified as being over-expressed in our study. Insecticide resistance through decreased cuticular penetration has been demonstrated in several insect species including the red flour beetle (*Tribolium castaneum*) cotton bollworm (*Helicoverpa armigera*) and German Cockroach (*Blattella germanica*) [27]–[29]. In addition, over-expression of two cuticular precursor genes has been reported in pyrethroid resistant *Anopheles gambiae* [30].

The potential role of reduced cuticular penetration in neonicotinoid resistance in *M. persicae* was supported by our further biological and biochemical studies. Firstly, artificial feeding assays with the neonicotinoid clothianidin showed that the LC_50_s for the susceptible clone and the resistant clone were 8 and 17 times lower than those obtained with topical applications, suggesting that there is a difference in penetration. Secondly, *in vivo* penetration assays using [^3^H] imidacloprid revealed a significant reduction in the penetration of insecticide through the cuticle in the resistant clone. Taken together, these results suggest that over-expression of CPs may also play a role in resistance to neonicotinoid insecticides in *M. persicae.* However, the exact role CPs play in cuticular thickening and reduced penetration remains unknown and clearly warrants further investigation.

Although resistance is an almost inevitable outcome of intensive insecticide use it is possible to minimize its effect by adopting resistance management strategies which aim to prevent, or more usually slow, the development and spread of resistance. Such strategies often include minimizing the use of certain pesticides and rotating the usage of compounds with different target sites. In this regard this study provides valuable insights into the molecular basis of detoxification and the resistance mechanisms characterized here are new additions to those described previously in *M. persicae* which include MACE, kdr, and increased production of esterases (see introduction). Guidelines on managing resistance in this pest have been published by the Insecticide Resistance Action Group (IRAG) [31] and highlight insecticides with alternative modes of action such as the anti-feedants pymetrozine and flonicamid that may not be affected by these existing mechanisms and therefore represent potential alternatives for control of this pest.

In summary, we have shown through bioassays that P450-mediated detoxification likely plays a substantial role in neonicotinoid resistance in *M. persicae* but that other secondary mechanism(s) may be involved. The resistant phenotype is associated with the over-expression of a single P450 gene and we show that this is due, at least in part, to gene amplification. Over-expression of cuticular proteins and reduced penetration of insecticide may make an additional contribution but there is no evidence of a role for target-site insensitivity in the clone investigated.

## Materials and Methods

### Aphid clones and rearing

4106A is an insecticide-susceptible clone originally collected from potatoes in Scotland in 2000. Clone 5191A was collected from tobacco in Greece in 2007 and has the FE4 variant of enhanced esterase at R3 levels and a ∼50 fold resistance to the neonicotinoid insecticide imidacloprid [13]. Clones 4255A and 5142B were collected in the UK from oilseed rape and sugar beet respectively. Clone 1200Q was collected from peach in Argentina. All *M. persicae* clones were originally established from a single parthenogenetic female and were reared asexually on Chinese cabbage leaves (*Brassica napus* L var *chinensis* cv Tip-Top) in small plastic box-cages maintained at 18°C under a 16:8 h light:dark regime. Aphids of similar age were transferred regularly to new boxes in order to rear them as distinct cohorts.

### Insecticides and synergists

Technical-grade insecticides (imidacloprid, clothianidin, thiamethoxam, thiacloprid) were purchased from Sigma (Sigma-Aldrich, UK) and technical piperonyl butoxide (PCP ‘Ultra’) was provided by Dr. Graham Moores. [^3^H] imidacloprid was either from a sample provided by Dr Ralf Nauen, Bayer CropScience (38 Ci/mmol) or purchased from American Radiolabeled Chemicals, Inc. (40 Ci/mmol).

### Topical bioassays

Ten adult aphids were placed on the abaxial surface of a Chinese cabbage leaf disk sitting on 1% agar in a plastic tub (3 cm diameter). The walls of the tub above the leaf were coated with Fluon to prevent escape. The aphids were allowed to settle for an hour before being dosed individually with 0.25 µl acetone containing the insecticide to be tested using a microapplicator (Burkard Manufacturing Ltd, UK). For the synergism bioassays, aphids were initially dosed with 0.1% PBO solution in acetone followed 5 h later by the insecticide dose. For the initial screening of the clones a diagnostic dose of 10 ppm imidacloprid solution was used while for full dose-response bioassays a range of concentrations was applied. Control aphids were dosed with 0.25 µl acetone. Three replicates were used for each concentration tested and the responses were assessed after 72 h at 18°C under a 16:8 h light:dark regime. Aphids that were dead or seriously affected were classed as ‘dead’ and the concentrations required to kill 50% (LC_50_ values) were calculated by probit analysis using POLO software (LeOra Software, Petaluma, CA). Resistance ratios were calculated relative to the susceptible clone 4106A and synergism ratios were calculated by dividing the LC_50_ to an insecticide alone by the LC_50_ of the synergised clone (pre-treated with PBO) to the same insecticide.

### Artificial feeding assays

The oral toxicity of imidacloprid, clothianidin and thiamethoxam was assessed using an artificial double-membrane feeding bioassay. Aphids (10 individuals) were placed in a vented plastic dish (4 cm diameter) and the opening was sealed with Parafilm. Aphids were then starved overnight before 0.4 ml sucrose (150 g litre^−1^) containing the appropriate insecticide concentration was pipetted onto the Parafilm and another layer stretched on top to form a sachet. In order to avoid false positive results due to the potential anti-feeding effect of the insecticide, a starvation control was set alongside the treatments which had no sucrose added. The dishes were covered with a yellow bag to enhance feeding activity and placed at 18°C under a 16:8 h light:dark regime. Mortality was scored after 48 h and data analysed as for topical application bioassays.

### Microarray

The microarray used in this study was designed using the Agilent eArray platform (Agilent Technologies) by the Georg Jander Lab and is based on a previously described array containing probes for >10, 000 *M. persicae* unigenes produced by Sanger sequencing [17] augmented with an additional 30, 517 probe set designed on EST unigene sequences identified in a 454 sequencing project [18]. The final slide layout consists of four arrays of 45, 220 60-mer probes produced by Agilent by *in situ* oligonucleotide synthesis.

Total RNA was extracted from four pools of ten 15 day-old adult aphids using Trizol following the manufacturer's instructions. Genomic DNA was removed by DNase I digestion using DNA-free DNase Treatment and Removal Reagent (Ambion). The quality and quantity of the RNAs were assessed by spectrophotometery (Nanodrop Technologies) and by running an aliquot on a 1.5% agarose gel. For the latter RNA was mixed with 1× loading buffer (95% formamide; 0.025% xylene cyanol; 0.025% bromophenol blue; 18 mM EDTA; 0.025% SDS), heated for 5 minutes at 65°C and briefly chilled on ice prior to loading. Two micrograms of each RNA was used to generate labelled cRNA, which was hybridized to arrays and these were washed and scanned as described in the Agilent Quick Amp Labeling Protocol (Version 5.7) [32]. The microarray experiment consisted of four biological replicates and for each of these, two hybridisations were done in which the Cy3 and Cy5 labels were swapped between samples for a total of eight hybridisations between resistant and susceptible clones.

Microarrays were scanned with an Agilent G2565CA scanner, and fluorescent intensities of individual spots were obtained using the Agilent Feature Extraction software with default Agilent parameters. The TM4 suite of software from the Institute of Genomic Research was used for all subsequent analysis [33]. Each Agilent feature extraction text file was transferred to TIGR Express Converter to generate TIGR MultiExperiment Viewer files (.mev) as output. Data normalization, filtering, and flip-dye consistency checks were performed using TIGR MIDAS. Briefly, spot intensity values over 300 were transformed to the log_2_ scale and data were normalized using locally weighted linear regression (lowess) with 33% of data used for smoothing and a cut-off of 0.01 [34]. Normalized data were then imported into the MultiExperiment Viewer (MeV) software and statistical analysis performed with the SAM (Significance Analysis of Microarrays) module applying a False Discovery Rate (FDR) of zero to detect significantly differentially expressed genes [35]. Genes identified by SAM and showing a transcription ratio >2 fold in either direction were considered to be differentially transcribed between the two strains. Microarray data were submitted to the Gene Expression Omnibus (GEO) database (series no. GSE20542).

### Quantitative RT–PCR

Quantitative RT-PCR was used to validate microarray data by examining the expression profile of selected genes. Primers were designed to amplify a fragment 90–150 bp in size and are listed in Table S2. Total RNA was prepared and DNase I treated as described and four micrograms was used for cDNA synthesis using Superscript III and random hexamers (Invitrogen) according to the manufacturer's instructions. PCR reactions (20 µl) contained 4 µl of cDNA (10 ng), 10 µl of SensiMix SYBR Kit (Bioline), and 0.25 µM of each primer. Samples were run on a Rotor-Gene 6000 (Corbett Research) using the temperature cycling conditions of: 10 minutes at 95°C followed by 40 cycles of 95°C for 15 seconds, 57°C for 15 seconds and 72°C for 20 seconds. A final melt-curve step was included post-PCR (ramping from 72°C–95°C by 1°C every 5 seconds) to confirm the absence of any non-specific amplification. The efficiency of PCR for each primer pair was assessed using a serial dilution of 100 ng to 0.01 ng of cDNA. Each qRT-PCR experiment consisted of three independent biological replicates with three technical replicates for each. Data were analysed according to the ΔΔC_T_ method [36], using the geometric mean of three selected housekeeping genes (actin, *para* which encodes the voltage gated sodium channel, and *ace*, which encodes acetylcholinesterase) for normalisation according to the strategy described previously [37].

### Determination of P450 gene copy number by quantitative PCR

Quantitative PCR was used to determine *CYP6CY3* gene copy number essentially as described above but using genomic DNA as the template. For this DNA from three pools of five 15 day-old adult aphids were extracted using the DNeasy Plant DNA Mini Kit (Qiagen) and using RNase A to remove contaminating RNA. DNA quality and quantity was assessed by spectrophotometery (Nanodrop Technologies) and by running an aliquot on a 1.5% agarose gel. The DNA was then diluted to 2.5 ng/ul and 4 µl used in PCR as detailed above. Data were analysed according to the ΔΔC_T_ method [36] and normalised independently using three genes, *para* (present in two copies in diploid insect genomes), *ace* (present in four copies in *M. persicae*) and actin where the exact gene copy number is unknown but is thought to be multi-copy [10].

### Rapid amplification of cDNA ends and amplification of full length cDNA from *CYP6CY3*

Five prime RACE was carried out using the RLM-RACE kit (Ambion) following the manufacturer's instructions. RACE was performed on RNA extracted from both the 4106A and 5191A *M. persicae* clones. The gene-specific primers for this purpose (Mpcyp6 mid r, Mpcyp6 mid r2, Mpcyp6 5′ outer R and Mpcyp6 5′ inner R) are detailed in Table S2. PCR products were recovered from agarose gels, cloned using the Strataclone PCR Cloning kit (Stratagene) and sequenced using the ABI BigDye Terminator Cycle Sequencing kit and T3/T7 primers. To verify the assembly the full length coding sequence of *CYP6CY3* was amplified by nested PCR using primers Full F1 and Full R1, followed by Full F2 and Full R2 (Table S1). PCR reactions (20 µl) contained 4 µl of cDNA (10 ng), 1.5 units of Pfu DNA polymerase (Promega) and 0.5 µM of each primer and were subjected to cycling conditions of: 2 minutes at 94°C followed by 35 cycles of 95°C for 30 seconds, 57°C for 30 seconds and 72°C for 4 minutes. PCR products were purified and cloned and sequenced as above or sequenced directly using primers Full F2, Full R2, Mpcyp6 mid r and Mpcyp6 mid r2 (Table S2).

### Membrane preparation and radioligand binding assays

Aphids of mixed ages (∼0.5 g) were homogenised in ice cold 10 mM phosphate buffer (pH 7.4) with 0.32 M sucrose, 1 mM EDTA, 1 µM leupeptin, 1 µg/ml pepstatin, 250 µM PMSF and 2 µl/ml protease inhibitor cocktail P-8340 (Sigma-Aldrich, UK) using a Polytron homogeniser. The homogenate was centrifuged for 10 min at 700 *g* (4°C) and the resulting supernatant passed through four layers of synthetic mesh before being re-centrifuged for 1 h at 105000 *g* (4°C). The pellet was resuspended in 10 mM phosphate buffer (pH 7.4) and the protein concentration measured using the DC protein assay (Bio-Rad laboratories) with bovine serum albumin as a standard. For the binding assay, 300 µg of membranes were incubated with 3 nM [^3^H] imidacloprid in a total volume of 300 µl. Non-specific binding was determined by blocking the nAChRs with 1 mM carbamylcholine and 1 mM nicotine prior to the addition of [^3^H] imidacloprid. The samples were incubated for 2 hours at 4°C on a shaker, filtered through Whatman GF/B filters pre-soaked in 0.5% polyethylenimine and washed with 10 mM phosphate buffer (pH 7.4) using a cell harvester (Brandel, Bethesda, MD). The assay was repeated three times for each clone.

### Sequence analysis of *M. persicae* nAChR subunits

Total RNA was isolated from individual 4106A and 5191A adult aphids using Trizol (Invitrogen) following the manufacturer's protocol. First strand cDNA was synthesised from total RNA (1 µg) using SuperScript II reverse transcriptase (Invitrogen) and oligo-dT primer (100 ng). PCR amplification was performed using PCR Master Mix (Promega) and gene-specific oligonucleotide primers designed to the published nAChRα1 (X81887), nAChRα2 (X81888), nAChRα3 (AJ236786), nAChRα4 (AJ236787) and nAChRβ1 (AJ251838) sequences (see Table S2). The cycling conditions were 2 min at 94°C followed by 35 cycles of 30 sec at 94°C, 30 sec at 50°C and 1 min at 72°C and a final extension step of 5 min at 72°C. Amplicons of the expected size were ethanol precipitated and sequenced directly in both directions using the BigDye mix (Applied Biosystems).

### Imidacloprid penetration assay

For the [^3^H] imidacloprid penetration assay three replicates of ten aphids each were used for each time point for both the susceptible (4106A) and resistant clone (5191A). Individuals of both clones were dosed with a sub-lethal dose of imidacloprid (0.14 ppm). A 0.25 µl drop of acetone containing 0.14 ppm [^3^H] imidacloprid was applied to each individual using a microapplicator (Burkard Manufacturing Ltd, UK). Samples (three replicates) from each clone were taken after 0.5, 1, 5, 10, 20 and 50 h to measure the degree of insecticide penetration. Aphids from each replicate were pooled in a tube and washed three times with 50 µl methanol. The three washes containing the imidacloprid on the cuticle were pooled together in a scintillation vial. The radiolabeled imidacloprid levels in the washes were measured by liquid scintillation spectrometry by adding 4 ml scintillation liquid (Ultima Gold, Perkin Elmer Inc. MA) over the methanolic extract, mixing and counting for 10 minutes. In addition, ten drops (0.25 µl) of the dosing solution was added to a glass filter paper, placed in 4 ml scintillation liquid and counted for the total number of counts expected per replicate. Results were expressed as a percentage of the total imidacloprid added to the treatment.

### Sequence analysis

Molecular mass and isoelectric point were predicted by Compute pI/Mw tool (http://us.expasy.org/tools/pi_tool.html). The N-terminal transmembrane anchor of deduced proteins was predicted by the TMHMM Server v. 1.0 (http://www.cbs.dtu.dk/services/TMHMM/). DNA and predicted protein sequences were assembled, analysed and aligned using the Vector NTI Advance 10 package (Invitrogen). The full P450 gene sequence identified in this study was named by Dr David Nelson (Department of Molecular Science, University of Tennessee, Memphis) in accordance with the P450 nomenclature committee convention [15].

## Supporting Information

Figure S1 An amino acid alignment of *Acyrthosiphon pisum* CYP6CY3 with five partial EST gene sequences encoding P450s identified by microarray with elevated expression in the insecticide resistant *M. persicae* clone 5191A.
(0.10 MB DOC)

Table S1 Genes identified by microarray analysis significantly differentially transcribed between the insecticide resistant *M. persicae* clone 5191A and the susceptible clone 4106A. Microarray array analysis identified 273 genes significantly differentially transcribed between the insecticide resistant clone 5191A and the susceptible clone 4106A. The full list of these genes along with Log2, calculated fold-change values and a description based on the closest BLAST hit is detailed.
(0.08 MB XLS)

Table S2 Sequences of primers used in this study. Primer sequences are listed along with the purpose for which they were used.
(0.03 MB XLS)
