# Supplementary material for: Amplification of a Cytochrome P450 Gene Is Associated with Resistance to Neonicotinoid Insecticides in the Aphid Myzus persicae
Source: PLoS Genet. 2010 Jun 24;6(6):e1000999. doi: 10.1371/journal.pgen.1000999 (PMC2891718; doi:10.1371/journal.pgen.1000999)
Supplement: Figure S1 — An amino acid alignment of Acyrthosiphon pisum CYP6CY3 with five partial EST gene sequences encoding P450s identified by microarray with elevated expression in the insecticide resistant M. persicae clone 5191A. (0.10 MB DOC) [file pgen.1000999.s001.doc]

**Supplementary Figure 1. An amino acid alignment of *Acyrthosiphon pisum* CYP6CY3 with five partial EST gene sequences encoding P450s identified by microarray with elevated expression in the insecticide resistant *M. persicae* clone 5191A.**
